# Targeted genome editing in acute lymphoblastic leukemia: a review

**DOI:** 10.1186/s12896-018-0455-9

**Published:** 2018-07-17

**Authors:** Adrián Montaño, Maribel Forero-Castro, Jesús-María Hernández-Rivas, Ignacio García-Tuñón, Rocío Benito

**Affiliations:** 1 IBSAL, IBMCC, University of Salamanca-CSIC, Cancer Research Center, Salamanca, Spain; 2 School of Biological Sciences (GICBUPTC Research group), Universidad Pedagógica y Tecnológica de Colombia, Boyacá, Colombia; 3 Department of Medicine, University of Salamanca, Spain, Department of Hematology, University Hospital of Salamanca, Salamanca, Spain; 4 IBMCC, CIC University of Salamanca-CSIC, University Hospital of Salamanca, Salamanca, Spain

**Keywords:** Acute lymphoblastic leukemia, CRISPR-Cas9, Genome editing

## Abstract

**Background:**

Genome editing technologies offers new opportunities for tackling diseases such as acute lymphoblastic leukemia (ALL) that have been beyond the reach of previous therapies.

**Results:**

We show how the recent availability of genome-editing tools such as CRISPR-Cas9 are an important means of advancing functional studies of ALL through the incorporation, elimination and modification of somatic mutations and fusion genes in cell lines and mouse models. These tools not only broaden the understanding of the involvement of various genetic alterations in the pathogenesis of the disease but also identify new therapeutic targets for future clinical trials.

**Conclusions:**

New approaches including CRISPR-Cas9 are crucial for functional studies of genetic aberrations driving cancer progression, and that may be responsible for treatment resistance and relapses. By using this approach, diseases can be more faithfully reproduced and new therapeutic targets and approaches found.

## Background

Acute lymphoblastic leukemia (ALL) is a malignant disorder originating from hematopoietic B- or T-cell precursors, characterized by marked heterogeneity at the molecular and clinical levels. Although a genetic event is known to occur in the majority of cases, and may be associated with outcome prediction, around 25–30% of pediatric and 50% of adult ALL patients have no defined genetic hallmarks of biological or clinical significance [1].

The development of new techniques of genetic editing such as TALENs or CRISPR-Cas9 has made it possible to produce powerful animal genetic models that recapitulate the cooperating oncogenic lesions affecting genes with an established role in the proliferation and establishment of the leukemic clone [2]. In ALL, some approaches have been oriented towards analyzing the targeting of transcriptional factors such as *PAX5*, which are involved in the pathogenesis of B-ALL, and *TAL1* and *LMO2*, which are highly deregulated in T-ALL [3–5]. Targeting gene fusion expression has also been addressed through genome editing systems, as with *MLL* and *AF4,* whose fusion is associated with poor prognosis and which mainly affects B-ALL infants [6]. Other genes have been modified to gain a better understanding of the mechanism of action of several drugs, for example, *BTK*, target of ibrutinib, *XPO1*, the target of KPT-8602, and *CB1* and *CB2*, the targets of dronabinol [7, 8]. However, genome editing techniques have gone a step further and they have been used with therapeutic and clinical approaches. Their use has facilitated the design of new therapies such as chimeric antigen receptors (CARs) and have allowed the study of genes involved in the evolution of pathogenesis [9, 10].

### Targeted genome editing in ALL

The development of next generation sequencing (NGS) techniques provided enormous amount of data to interpret, which generated the need to translate those data into functionally and clinically relevant knowledge that enable investigators to determinate how genotype influences phenotype. In the past decade, the integration of genome editing systems enables investigators to directly manipulate virtually any gene in a diverse range of cell types and organisms [11].

**Fig. 1:**
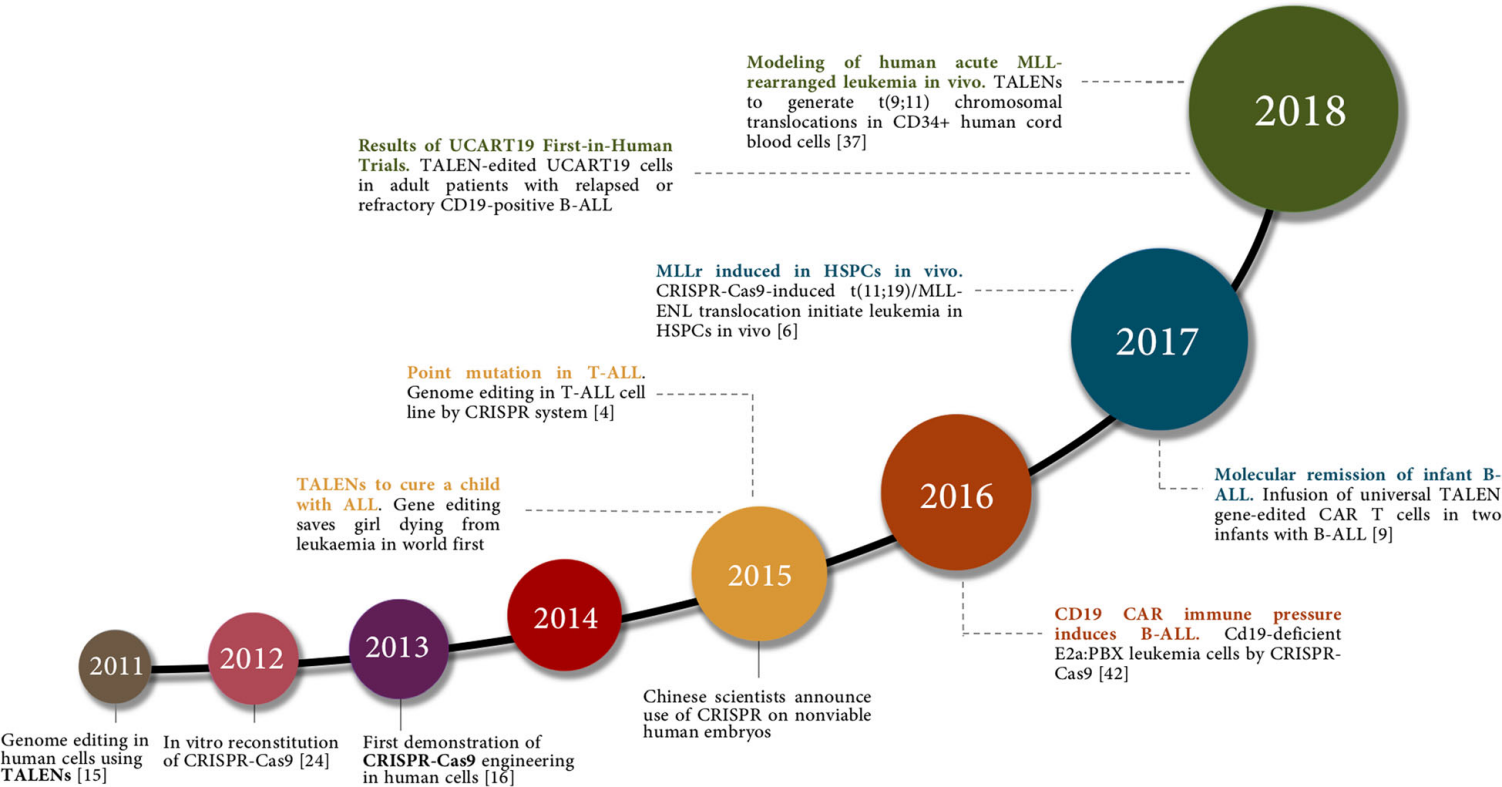
Timeline of genome editing engineering in ALL

**Fig. 2:**
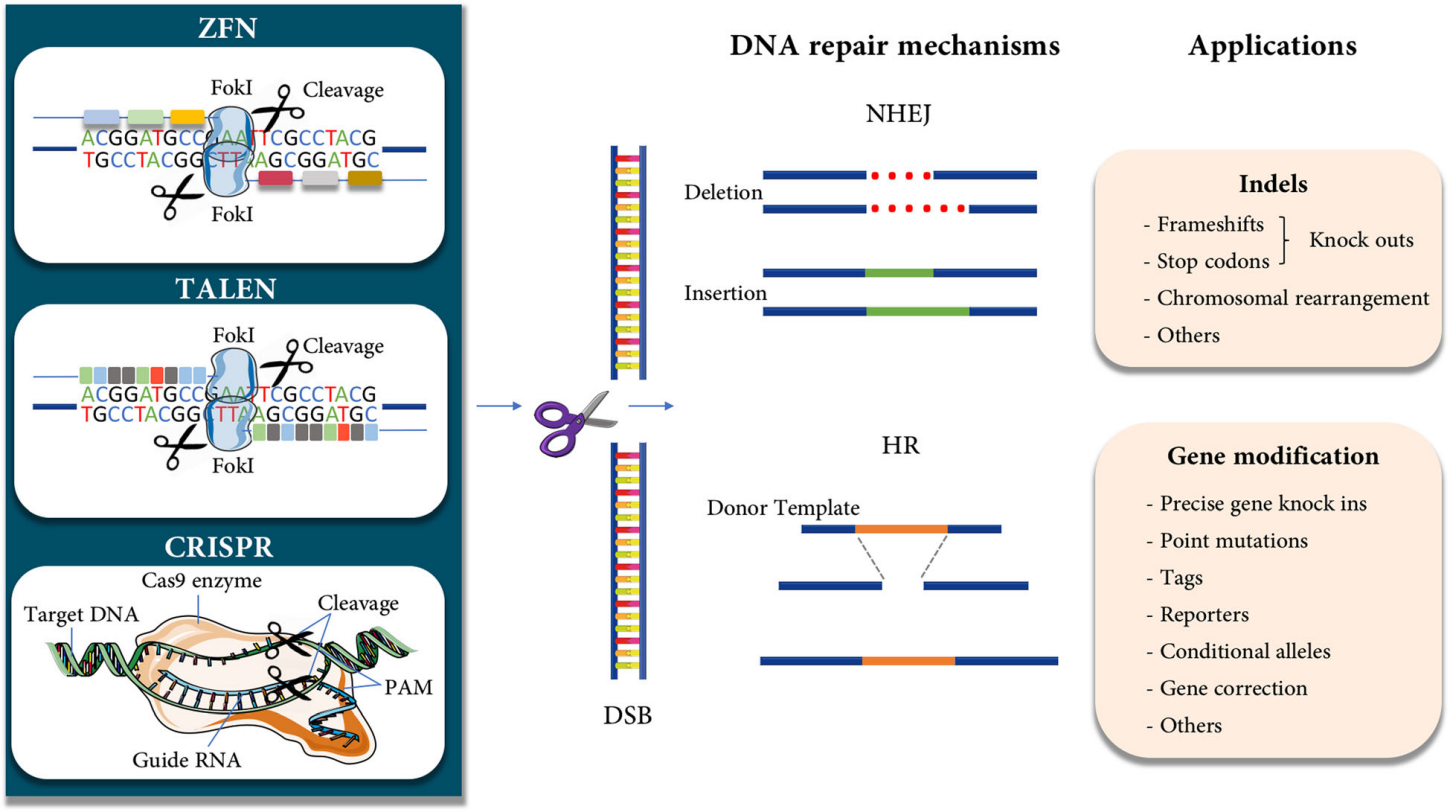
The nuclease genome editing technologies in ALL. The three most commonly used types of nucleases include programmable nucleases like Zinc Finger Nucleases (ZFNs), transcription activator-like effector nucleases (TALENs) and CRISPR systems (Clustered Regularly Interspaced Short Palindromic Repeats). These nucleases were able to induce double-strand breakgs (DSBs) in the target followed by the activation of DNA repair mechanisms [30]. On induction of double-stranded breaks or nicks at targeted regions, repairing is done by either Non-homologous end joining (NHEJ) or Homologous recombination (HR) pathway. NHEJ is an error prone repair mechanism where joining of broken ends takes place, which generally results in heterogeneous indels (insertions and deletions) whereas HR is a precise repair method in which homologous donor template DNA is being used in repair DNA damage target site. HR is the ideal strategy for generating knock in models [81, 82].

Genome editing system is based in the use of engineered nucleases composed of sequence-specific DNA-binding domains fused to a non-specific DNA cleavage module [12, 13]. These chimeric nucleases inducing DNA double-strand-breaks (DSBs) that stimulate the cellular DNA mechanisms, including error-prone non-homologous end joining (NHEJ) and homologous recombination (HR) [14]. Several approaches have been used in the last years as genome editing technologies (Fig. 1). The combination of simplicity and flexibility has hurtled zinc-finger nucleases (ZFNs), transcription activator-like effector nucleases (TALENs) and short palindromic repeats interspersed with regular intervals (CRISPR) to the forefront of genetic engineering (Fig. 2) [11].

ZFNs and TALENs were first used to generate knock-out rats in 2009 and 2011, respectively [15, 16]. TALENs system was first used in human cells in the same year [17]. CRISPR-Cas9 system, discovered as part of the prokaryotic adaptive immune system at the end of 1980s [18], was introduced some years later. This was proposed as a genetic modification system in 2005 [19] but was not tested until 2012 [20].

CRISPR-Cas9 is presented as a faster, cheaper, simpler system with the potential for multiplex genome editing [21]. The method is based on generating a directed cut in the double strand of DNA by the Cas9 nuclease. This is driven by a single 20-nucleotide RNA strand, which marks the exact breakage point. After DNA cutting, the DNA repair machinery of the host cell leads to repair errors and thereby promote a modification of the original sequence by a mutation such as an insertion, deletion or inversion, among others [22]. Based on CRISPR-Cas9 system, CRISPR interference (CRISPRi) and CRISPR activation (CRISPRa) emerged. CRISPRi uses a catalytically inactive version of Cas9 (dCas9) lacking endonucleolytic activity in combination with an sgRNA designed with a 20-bp complementary region to any gene of interest to silence a target gene [23]. While CRISPRa uses fusions of dCas9 to activator domains to activate gene expression [21].

Genome editing strategies have been used in several organisms, including *Drosophila* [24], *Caenorhabditis elegans* [25], zebrafish [26], mouse [27], rat [28], plants and humans [21] and has allowed a large number of functional studies to be carried out, based on the generation of animal and plant models. The use of genetically modified cell lines and animal models to understand the functions of genes and their pathogenesis in diseases conditioned by molecular genetics could be of help and provide insights to better understand cancer. The method used until now to generate these animal models, especially mice, is tedious and time-consuming, but CRISPR-Cas9 makes the procedure easier and more efficient [29].

Genome editing technologies, such as CRISPR-Cas9, have already been applied to the study of many diseases, including hematological diseases [30]. As exemplified by some very recent studies in Fanconi anemia (FA), a genetic DNA repair-deficient human disorder that results from mutations in FA genes [31] or the study of BCR-ABL oncogene in chronic myeloid leukemia [32]. Specifically, most of the genetic modification studies in ALL have been with CRISPR-Cas9, more than 20 articles since 2015. The vast majority had the purpose of knocking out genes, either by introducing mutations, insertions or deletions.

An overview of recent studies in genome editing in ALL is summarized in Table 1. Some of the most relevant studies included in the table are detailed below.

**Table 1:** Applications of genome editing systems in ALL

	Outcome	Target Gene	SSN Technique	Modification Type	Cell type	Reference
Targeting Transcriptional factors	Point mutation (insertion/deletion)	***TAL1***	CRISPR-Cas9	HR	PEER (in vitro)	[4]
	Repress expression	***PAX5***	CRISPR-a	NA	Patient-derived pre-B ALL cells (in vitro)	[3]
	Knock out	***NR3C1***	CRISPR-Cas9	NHEJ		
		***TXNIP***				
		***CB2***				
	Knock out	***LMO2***	CRISPR-Cas9	NHEJ	PF-382 (in vitro)	[5]
Targeting gene fusion expression	Chromosomal rearrangement	**MLL/AF4**	TALEN	NHEJ	K562, HSPCS (in vitro)	[42]
		**MLL/AF9**				
	Knock in	**MLL/AF4 AF4/MLL**	CRISPR-Cas9	HR	HEK293 (in vitro)	[46]
	Chromosomal rearrangement	**MLL/ENL**	CRISPR-Cas9	NHEJ	HSPCS (in vitro / in vivo, xenograft)	[6]
	Chromosomal rearrangement	**MLL/AF9**	TALEN	NHEJ	CD34+ human cord blood (in vivo, xenograft)	[43]
		**AF9/MLL**				
	Knock in	**ETV6/RUNX1**	CRISPR-Cas9	HR	MIFF3 hIPSCs (in vitro)	[40]
Drug targts discovery and therapy	Knock out	***CB1***	CRISPR-Cas9	NHEJ	Jurkat (in vitro)	[7]
		***CB2***				
	Knock out	***BTK***	CRISPR-Cas9	NHEJ	RCH-ACV (in vitro / in vivo, xenograft)	[8]
		***BLK***				
	Knock in	***XPO1***	CRISPR-Cas9	HR	HL-60, Jurkat, K-562, and MOLT-4 (in vitro / in vivo, xenograft)	[57]
	Knock out	***ABCB1***	CRISPR-Cas9	NHEJ	HALO1 (in vitro)	[59]
Modification of CARs	Knock out	***CD19***	CRISPR-Cas9	NHEJ	NALM6, 697 (in vitro / in vivo, xenograft)	[65]
	Knock out	***CD19***	CRISPR-Cas9	NHEJ	Murine leukemia cell lines E2a:PBX (in vitro / in vivo, xenograft)	[83]
		***PAX5***				
		***EBF1***				
	Knock out	***TRAC***	CRISPR-Cas9	HR	NALM6 (in vitro / in vivo, xenograft)	[63]
	Knock in	***CD19***				
	Knock out	***CD52***	TALEN	NHEJ	Two infants (in vivo)	[9]
		***TCR ab***				
	Knock out	***TCR b***	CRISPR-Cas9	NHEJ	PBMC (in vitro)	[84]
	Knock out	***CD7***	CRISPR-Cas9	NHEJ	T cell lines (in vitro / in vivo, xenograft)	[85]
		***TRAC***				
Evolution of pathogenesis	Knock out	***CASD1***	CRISPR-Cas9	NHEJ	HAP1 (in vitro)	[70]
	Knock out	***RIP1***	CRISPR-Cas9	NHEJ	Patient-derived ALL cells (in vitro / in vivo, xenograft)	[75]
	Knock out	***CXCR4***	CRISPR-Cas9	NHEJ	NALM6 (in vitro / in vivo, xenograft)	[10]
Others	Knock out	***FLT3***	TALEN	NHEJ	K562 (in vitro)	[86]
	Knock out screen	NA	CRISPR-Cas9	NHEJ	NALM6 (in vitro)	[87]
	Knock out screen	NA	CRISPR-Cas9	NHEJ	MV4,11 (in vitro)	[88]
	Knock out	***NUDT15***	CRISPR-Cas9	NHEJ	Mouse (in vivo)	[89]
	Knock out	***DCK***	CRISPR-Cas9	NHEJ	KOPN41 (in vitro)	[90]
	Knock out	***PTCH1***	CRISPR-Cas9	NHEJ	Zebrafish embryos (in vivo, xenograft)	[91]

This table shows the main genetic editing studies carried out in ALL, classified according to the target. The different columns indicate: the outcome of edition, the target of edition (highlighted in bold), the technique used, the type of modification, the cell type and the reference

### Targeting transcriptional factors

Deregulation of Transcription factors (TFs) is a common mechanism in the pathogenesis of human cancer, in particular in leukemia cells, genes encoding TFs are often amplified, deleted, rearranged via chromosomal translocation, or subjected to point mutations that result in a gain- or loss-of-function. Consequently, targeting of TFs can be highly effective in treating ALL. TFs such as *PAX5* and *IKZF1* were altered in nearly 80% of patients with B-ALL [33, 34]. These alterations affected glucose metabolism and energy supply, whereby the transcription factors act as metabolic repressors by limiting the amount of ATP available. A CRISPR-Cas9-based screen of *PAX5* and *IKZF1* transcriptional targets identified some target genes such as *NR3C1*, *TXNIP* and *CB2* as central effectors of B-lymphoid restriction of glucose and energy supply and therefore new targets for treating B-ALL [3].

In human T-cell acute lymphoblastic leukemia (T-ALL) cells, a CRISPR-Cas9 editing tool was used to disrupt *TAL1* (*SCL*) [4] or *TRIB1* (*TRB1*) [35] genes to delineate their biological functions. *TAL1* is one of the oncogenes most frequently deregulated in T-ALL [36]. This deregulation is produced by t (1;14) (p34;q11) (1–2%) or *SIL(STIL)-TAL1* deletions (del(1)(p32)) (15–20%), although there is still a large group of patients in whom the gene is deregulated but not altered. Epigenetics must therefore play an important role in these patients [37]. CRISPR-Cas9 was used in a cell line to reproduce two known alterations in *TAL1* (insertion and deletion) and it was observed how these alterations triggered their expression. Furthermore, a change in methylation acetylation of H3K27 was observed, suggesting a causal relationship between mutagenesis, epigenetic modulation and expression of *TAL1* [4].

*LMO2* is another gene deregulated in T-ALL. It is a potent oncogene that is essential for the formation of a large transcriptional complex in which genes such as *TAL1*, *LDB1*, *GATA1*, *GATA2*, *GATA3*, *RUNX1*, *ETS1*, and *MYB* intervene. Furthermore, its overexpression has been associated with the development of T-ALL. However, the reasons why this gene is overexpressed remain unclear, because few mutations have been described. Mutations targeted to the non-coding region of *LMO2* were introduced in a T-ALL cell line by CRISPR-Cas9 and proved to be a possible cause of the deregulation of *LMO2* expression [5].

### Targeting gene fusion expression in ALL with chromosomal rearrangements

As indicated above, chromosomal translocations are very frequent in ALL and can be used to stratify the risk of ALL patients. It is well known that *MLL* rearrangements occur in a small percentage of B-ALL patients, where they are associated with very poor prognosis. Several studies have proposed that *MLL* rearrangements are an initiating event in leukemic transformation, unlike ETV6-RUNX1 and BCR-ABL translocations, in which second events are necessary to initiate leukemia [33, 38, 39]. This was demonstrated by Enver T’s group, who used a homologous recombination knock-in approach by CRISPR-Cas9 to introduce the cDNA encoding of *RUNX1* exons 2–8 into the native *ETV6* locus of hIPSC. ETV6-RUNX1 expression induced a partial block of the maturation of B lymphocytes, at which time the second events required for leukemia development occurs [40].

Matthew Porteus’s group wanted to test the oncogenic potential as initiator event of the *MLL* translocations, and for this purpose, they generated the MLL-AF4 and MLL-AF9 translocations by genetic modification in primary hematopoietic stem and progenitor cells (HSPCs). This strategy was based on previous studies that demonstrated that the double-stranded DNA breakage at specific positions of two chromosomes could lead to translocation [41]. They used TALENs to generate cuts directed at specific positions of MLL-AF4 and AF9*,* based on the breakage points described in patients. In vitro, the cells that acquired the translocation showed a proliferative advantage over the others but were not able to transform completely because they eventually disappeared from the culture [42].

Shortly after, Heckl D.’s group showed strong evidence for the formation of true t (11;19)*/*MLL-AF9 translocations in vitro and in vivo by CRISPR-Cas9. No full transformation was observed in liquid cultures or methylcellulose-based in vitro assays using CD34+ HSPC, while in vivo assays demonstrated that endogenous t (11;19) can initiate a monocytic leukemia-like phenotype. This study is in line with the Matthew Porteus’s study, which emphasizes the importance of environmental cues for the oncogenic transformation in *MLL*r leukemias [6].

More recently, Stanford’s group managed to generate t (9;11) chromosomal translocations encoding MLL-AF9 and reciprocal AF9-MLL fusion products in CD34+ human cord blood cells by TALENs. Transplantation of these cells into immune-compromised mice induced myeloid leukemias with absence of secondary lesions studied by targeted exome sequencing and RNAseq [43].

The prevailing theory is that *MLL* rearrangements occur in the uterus due to exposure to certain chemicals during pregnancy that cause errors in DNA repair, as has been demonstrated in vitro and in vivo [44, 45]. The group of Pablo Menéndez examined how it affected the expression of the fusion protein in repairing DNA damage. To this end, MLL-AF4 protein and its reciprocal, AF4-MLL were induced in the AAVS1 locus of the HEK293 cell line by CRISPR-Cas9. They subsequently induced DNA damage by exposing the cells to etoposide and ionizing radiation (IR), with no differences in repair between WT cells and those expressing proteins. Thus, they demonstrated that the expression of the fusion proteins caused by *MLL* rearrangements, did not influence susceptibility to DNA damage or repair mechanisms [46].

### Drug targets discovery and therapy

The targets against which a drug acts must be identified and combined with the data provided by the NGS. This may sometimes identify patients with mutations in genes associated with some type of resistance. It can also help to generate other new drugs, when there is prior knowledge of the altered pathway we wish to attack. For example, ibrutinib has recently been proposed for the treatment of pre-BCR and *TCF3*-r-positive cases. Ibrutinib is an inhibitor kinase targeted to those ALL subtypes with affected BCR signaling. In order to understand the mechanism of action of ibrutinib in this ALL subtype, Bruton tyrosine kinase (*BTK*) KO, B lymphocyte kinase (*BLK*) KO and *BTK / BLK* KO cells have been generated by CRISPR-Cas9 [8].

The importance of *BTK* in the pathogenesis of chronic lymphocytic leukemia, diffuse large B-cell lymphoma, and other mature B-cell malignancies is well established [47–49], while there is less information about the role of *BTK* in ALL. Early studies reported unaltered levels of *BTK* in childhood ALL cells, whereas frequent *BTK* deficiency due to aberrant splicing was reported later [50, 51]. *BLK* and *BTK* were the only kinase genes overexpressed in this subtype of ALL, as revealed by arrays [52]. Only the elimination of the expression of both kinases managed to reduce the proliferation in a similar way to ibrutinib. However, these should not be the only targets of ibrutinib since the decrease in proliferation was still greater when the drug was used. To confirm that *BTK* and *BLK* were actually drug targets, ibrutinib was tested in cell lines generated with KO genes. This indicated that ibrutinib requires the presence of both kinases for maximum effectiveness [8].

In a subsequent study, Thomas Vercruysse and coworkers focused on exportin 1 (*XPO1*). *XPO1* plays an important role in the transport through the nucleus of cycle regulatory proteins and tumor suppressor proteins, among others. The overexpression of this gene is associated with several types of cancer, and with poor patient outcome [53, 54]. *XPO1* inhibitors act by binding to the reactive cysteine residue located at position 528, preventing the export of charged proteins to the cytoplasm [55, 56]. To verify that the drug binds specifically to act against *XPO1,* a point mutation was inserted at residue 528 by CRISPR-Cas9. When this occurred, the drug was not able to act, and the cells became resistant. Therefore, this study demonstrated that the drug is highly specific to *XPO1* and is potent against ALL [57].

More recently, Dronabinol (Tetrahydrocannabinol, THC), a US Food and Drug Administration-approved cannabinoid receptor (CNB) agonist for the treatment of chemotherapy-induced nausea and vomiting, was found to induce apoptosis in acute leukaemia cells, as evidenced by the abrogation of pro-apoptotic effects of CRISPR-mediated knockout of *CB1* or *CB2* following THC treatment [7, 58].

Furthermore, new drugs are being proposed as an alternative to current therapy. An example is Carfilzomib (CFZ), as a substitute of proteasome inhibitor Bortezomib (BTZ), who demonstrated favorable clinical outcomes for refractory childhood ALL. CFZ showed significantly higher activity than BTZ in vitro, except for the P-glycoprotein-positive t (17;19) ALL cell lines. Takahashi et al. generated a knock-out of *ABCB1*, who codes for P-glycoprotein, by genome editing with a CRISPR-Cas9 system and sensitized P-glycoprotein-positive t (17;19) ALL cell line to CFZ [59].

### Modification of CAR

Chemotherapy and/or radiotherapy have been standard treatments for ALL to date. However, immunological therapies have gained importance. These work by harnessing the immune system of patients to fight the disease. One example is chimeric antigen receptors (CARs), which are proteins genetically engineered to allow T cells to recognize a specific antigen in tumor cells. It had already been proposed as a standard therapy for ALL patients in 2013 by Rosenberg. In this case, the CARs were directed against CD19, an antigen of B cells [60]. Its efficacy had already been demonstrated in cases of refractory or relapsed ALL [61, 62].

CRISPR-Cas9 may be key to carry out this genetic modification. This was demonstrated by Michel Sadelain’s group. The strategy followed was the combination of knock-out and knock-in. On the one hand, they interrupted the TRAC locus, and on the other, they added a CAR directed to CD19, inserting it in the AAVS1 locus. hey compared responses to CD19 antigens from these cells with those from others in which CAR had been randomly integrated. In this way, they were able to demonstrate that targeted CAR integration under the control of endogenous regulatory elements is much more effective, reduces tonic signaling, avoids the differentiation and accelerated depletion of T cells, and increases the therapeutic potential of these cells [63].

Paul Veys’s group demonstrated the use of TALEN-modified T lymphocytes in two infants with refractory B-ALL. They generated universal T-cells against CD19 (CAR19), targeting the TALENs against the T-cell receptor (TCR) and simultaneously transfecting with non-human leukocyte donor cell antigens. As treated cells, they disrupted the CD52 gene, the target of the drug alemtuzumab, by TALEN, and also disrupted the expression of the αβ T cell surface receptor (TCR αβ). This minimized the risk of graft-versus-host disease (GVHD). The newborns were treated with lymphoplasty, chemotherapy and anti-CD52 serotherapy before infusion of CAR19. The results were very positive, yielding remissions within 28 days before allogeneic stem cell transplantation [9].

Despite the good results with CAR19 therapy, 10–20% of treated patients suffer relapses due to partial loss of the CD19 epitope [61, 64]. Andrei Thomas-Tikhonenko and his group have provided evidence that epitope loss is closely linked to alterations in exon 2 of CD19, detected in some samples from patients with relapses. These alterations include frameshift-type mutations and the total loss of the exon, resulting from an alternative splicing event that encodes a deficient isoform of exon 2. To assess the relevance of the detected isoforms, they eliminated CD19 expression by CRISPR-Cas9 from ALL cell lines, and then reconstituted them with different isoforms. They observed that the depleted isoform of exon 2 was located mostly in the cytosol, which could explain its mechanism of escape in front of CAR19. Thus, these deleterious mutations and the selection of isoforms resulting from alternative splicing could be the cause of this mechanism of resistance [65].

### Evolution of pathogenesis

Although there have been great advances in the treatment and cure of ALL, there is still a large group of patients who experience relapses, persistent minimal residual disease, and drug resistance, and who ultimately have a poor prognosis [66, 67]. Efforts have therefore focused on trying to understand why these resistances occur, to counteract them, and to look for new, more personalized drugs that avoid resistance.

In ALL, survival and drug resistance of lymphoblasts critically depend on 9-O-acetylation of sialic acids (Sias) [68, 69]. Baumann AM et al., generated a *CASD1* knock-out cells by CRISPR-Cas9-mediated genome editing and demonstrated that *CASD1* is essential for 9-O-acetylation [70].

Second mitochondrial-derived caspase-activators (SMACs) act by inhibiting inhibitors of apoptosis proteins (IAPs). One of the possible causes of resistance is revealed by the action of these proteins, which act to counteract the effects of drugs. These are also overexpressed in many types of cancer [71, 72]. The main mechanism of action of IAPs is the inhibition of apoptosis through proteins such as caspases [73] or receptor interaction of protein kinase 1 (*RIP1*)*,* a potent activator of death [74]. In this study, they set out to demonstrate that SMAC acted by reactivating apoptosis of these cells, mediated by *RIP1.* They used CRISPR-Cas9 system to knock out this gene in vivo in xenograft models, and thereby eliminate its expression. The results showed that *RIP1* was necessary for the induction of cell death by SMAC [75].

CXCR4 encodes a membrane receptor whose function is to attract and confine the stromal cells of the bone marrow stromal cells (BMSCs). This interaction with BMSCs gives B cells a degree of protection, associated with increased survival, resistance to treatment, relapse and worse prognosis [76, 77]. *CXCR4* is highly expressed in B-ALL cells and has also been correlated with poor patient outcome [78]. Inhibitors of *CXCR4* have already been examined in the preclinical setting, in vitro and in vivo [79, 80] and may be *CXCR4* antagonists or agonists. To test whether the efficacy of these compounds was due to the inhibition of *CXCR4* and not to their own activity as agonists, they generated a B-ALL cell line with *CXCR4* knock-out by CRISPR-Cas9. They demonstrated that the agonistic activity of *CXCR4* antagonists did not affect antitumor activity. In addition, in vivo *CXCR4* knock-out models reduced the burden of leukemia and disease progression. In this way, the importance of *CXCR4* in the pathogenesis of B-ALL and in its use as a therapeutic target to fight drug resistance is demonstrated [10].

## Conclusions, challenges and future directions

Genome editing technologies have already demonstrated its potential to study molecular biology and pathogenesis of the genetic aberrations in ALL, in vitro and in vivo.

From a future perspective, the development of the genomic editing tools could also help to the generation of murine models of leukemias that resemble the human disease. In this sense, multigenic nature of the disease entails great difficulties. In the case of ALL, murine models based on a single alteration have failed, at least in part, to fully develop the disease. Combining several of the gene alterations found in patients in a murine model, we could approach to the real pathological conditions, giving rise to a more efficient model for the investigation of this type of tumors. Until recently, to generate an animal model with several genetic alterations was a long and expensive process, however, tools such us, CRISPR-Cas9, will allow introducing multiple mutations in a single step. Thus, it will be possible to generate, in short periods of time, more complex animal models that allow us to simulate more faithfully the conditions that occur in patients, providing the appropriate platform to study and to develop new therapeutic strategies.

Furthermore, in the clinic, genome editing systems could facilitate the rapid screening of new drugs and will promote the development of personalized medicine, connecting genomics, disease phenotypes and therapeutic goals. The use of these technologies will broaden our understanding of the mechanism of action of these novel drugs and enable the identification of novel mechanisms of acquired resistance to pathway target therapeutics. However, translating genome editing technologies to the clinical setting requires two main concerns to be addressed: the safety and efficacy of treatments. The off-target effect remains one of the major obstacles of this technology. Researches will need to improve our genetic tools in order to eliminate any off-target effects and to improve the gene edition efficiency in the future. Despite this, genome editing offers new opportunities for tackling diseases such as ALL that have been beyond the reach of previous therapies.

## Abbreviations

ALL: Acute lymphoblastic leukemia
AML: Acute myeloid leukemia
BCR: B-cell receptor signaling
BMSCs: Bone marrow stromal cells
CARs: Chimeric antigen receptors
CRISPR: Short palindromic repeats interspersed with regular intervals
DSBs: Double-strand breaks
HiPSC: Human induced pluripotent stem cell
HR: Homologous recombination
HSPCs: Hematopoietic stem and progenitor cells
IAPs: Inhibitors of apoptosis proteins
IR: Ionizing radiation
MMEJ: Micro-homology-mediated end-joining
NGS: Next generation sequencing
NHEJ: Non-homologous end-joining
PBMC: Peripheral blood mononuclear cell
Sias: Sialic acids
SMACs: Second mitochondrial-derived caspase-activators
TALENs: Transcription-activating type nucleases
TCR: T-cell receptor
THC: Tetrahydrocannabinol
ZFNs: Zinc finger nucleases
